# Doxycycline-containing glass ionomer cement for arresting residual caries: an *in vitro* study and a pilot trial

**DOI:** 10.1590/1678-7757-2017-0116

**Published:** 2018-04-18

**Authors:** Aline Rogéria Freire de Castilho, Cristiane Duque, Paula Fernanda Kreling, Jesse Augusto Pereira, Andreia Bolzan de Paula, Mario Alexandre Coelho Sinhoreti, Regina Maria Puppin-Rontani

**Affiliations:** 1 Universidade Estadual de Campinas, Faculdade de Odontologia de Piracicaba, Departamento de Odontologia Infantil, Piracicaba, SP, Brasil; 2 Universidade Estadual Paulista, Faculdade de Odontologia de Araçatuba, Departamento de Odontologia Infantil e Social, Araçatuba, SP, Brasil; 3 Universidade Estadual de Campinas, Faculdade de Odontologia de Piracicaba, Departamento de Odontologia Restauradora, Piracicaba, SP, Brasil

**Keywords:** Glass ionomer cements, Dental caries, Antimicrobial Agents, Biomaterials, Clinical trial

## Abstract

In a previous study, we demonstrated that the incorporation of doxycycline hyclate (DOX) into resin-modified glass ionomer cement (RMGIC) inhibited important cariogenic microorganisms, without modifying its biological and mechanical characteristics. In this study, we keep focused on the effect of that experimental material as a potential therapy for arresting residual caries by analyzing other *in vitro* properties and conducting a pilot clinical trial assessing the *in vivo* effect of DOX-containing RMGIC on residual *mutans streptococci* after partial carious removal in primary molars. Specimens of the groups RMGIC (control); RMGIC + 1.5% DOX; RMGIC + 3% DOX; and RMGIC + 4.5% DOX were made to evaluate the effect of DOX incorporation on surface microhardness and fluoride release of RMGIC and against biofilm of *Streptococcus mutans.* Clinical intervention consisted of partial caries removal comparing RMGIC and RMGIC + 4.5% DOX as lining materials. After 3 months, clinical and microbiologic evaluations were performed. Data were submitted to ANOVA/Tukey or Wilcoxon/Mann-Whitney set as α=0.05. Fluoride release and surface microhardness was not influenced by the incorporation of DOX (p>0.05). There was a significant reduction of S. *mutans* biofilm over the material surface with the increase of DOX concentration. After clinical trial, the remaining dentin was hard and dry. Additionally, *mutans streptococci* were completely eliminated after 3 months of treatment with RMGIC + 4.5% DOX. The incorporation of DOX provided better antibiofilm effect, without jeopardizing fluoride release and surface microhardness of RMGIC. This combination also improved the *in vivo* shortterm microbiological effect of RMGIC after partial caries removal.

## Introduction

In developing countries, the prevalence of untreated dental caries is very high. In Brazil, the report from the Brazilian Oral Health Survey (SBBrasil 2010) showed that 48.2% of 5 years-old children have untreated dental caries or almost more than half of the population in this age group had at least one deciduous tooth affected by dental caries^1^. The reasons for the lack of treatment are multiple, such as financial problems, lack of oral health services, pain and fear barriers, and dependency of conventional oral care^2^. Alternative proposals to the complete caries removal technique have been investigated for permanent and deciduous teeth^3^. Scientific evidences have revealed that incomplete caries removal for teeth with deep cavities preserves a maximum of dental structure and reduces the possibility of pulp exposure^3^ ^−^ ^4^. Besides being clinically effective in retaining teeth and their vitality for longer, partial caries removal was also considered cost-effective, with significantly reduced long-term costs^5^.

Although encouraging clinical success rates have been reported in several studies^6^ ^−^ ^11^ and stated in some systematic reviews and meta-analysis^2^ ^,^ ^12^, there is still some resistance whether intentionally leaving carious dentin behind is safe, which could cause the failure of the dental treatment. Therefore, by using an effective antibacterial incorporated with lining material it must be possible to solve problems such as the cariogenic microorganisms remained after partial caries removal and consequently avoid caries progression and injuries to the pulp with no costs and less discomfort to the patient^13^ ^−^ ^18^. Even the literature indicates to leave residual carious dentin; the use of an antibacterial material over dentin layer would leave dentists more comfortable.

Doxycycline hyclate (DOX), a derivative of the tetracycline family, has been widely used in the treatment of various infectious diseases in Dentistry, including periodontal and root pathologies^19^ ^,^ ^20^. Based on study conducted thus far, we support the efficacy of a resin-modified glass-ionomer cement liner containing up to 4.5% DOX against selected cariogenic microorganisms, keeping original biological and mechanical characteristics of the cement^16^. Clearly, DOX exerts biological functions totally independent of its antibacterial properties, since this chemical agent blocks dentin proteolysis^21^. This is an interesting property for a substance, considering that caries progression is not only dependent on the bacterial activity, but is also related to the release of dentin matrix metalloproteinases^22^.

Our research group has focused on therapeutic approaches for caries using agents with specific activities against *Streptococcus mutans.* Therefore, based on early positive results concerning the mechanical properties of the glass ionomer cement and DOX association^16^ developed by the same research group, this study intended to provide more information about physical, antibacterial, and clinical properties of that experimental liner material. Then, we aimed to compare the *in vitro* microhardness, fluoride release, and antibiofilm properties of a DOX-containing RMGIC, besides to analyze the *in vivo* efficacy of that material on residual mutans streptococci after partial carious removal in primary molars.

## Materials and methods

The resin-modified glass ionomer cement (RMGIC) Fuji Lining LC (FLLC, batch #0710021, GC Corporation, Tokyo, Japan) was modified by the addition of 1.5%; 3.0%, and 4.5% doxycycline hyclate (D9891 Sigma-Aldrich Steinheim, Germany), according to the methodology described in previous studies^15^ ^,^ ^16^ ^,^ ^23^. All experiments were conducted in triplicate, in three independent assays.

### Specimen preparation for *in vitro* assays

Specimens from three experimental groups (RMGIC-containing 1.5, 3 or 4.5% DOX) and one control group (RMGIC) were prepared for surface microhardness (n=9), fluoride release (n = 9), and antimicrobial properties tests (n=9). RMGIC was mixed by agglutination of powder to liquid associated with or without 1.5, 3.0, and 4.5% DOX and then the mixture was placed with Centrix syringe (Centrix Inc., Shelton, USA) into cylindrical moulds (2 mm high × 5 mm diameter)^16^. The specimens were then exposed to a light source (Curing Light XL3000, 3M ESPE), with 410 mW/cm^2^ of light intensity for 30 s. Finally, they were stored in distilled water for 24 h at 37°C before being tested.

### Biofilm assays

A new proposal of biofilm assay was used in this study based on previous methodologies^24^ ^,^ ^25^. The objective was to evaluate the biofilm cellular viability on the RMGIC surface. Specimens were placed in 24-well plates with 2 μL *Streptococcus mutans* (ATCC 25175) suspension in 2 mL of BHI supplemented with 1% sucrose for 24 h at 37°C in 5% CO_2_. Then, specimens were washed in physiological solution (0.9% NaCl; 1 mL), inserted separately into microtubes containing 500 μL of NaCl, and sonicated in ultrasonic cell disruptor (XL; Misonix Inc., Farmingdale, NY, USA), according to the parameters used by Brighenti, et al.^26^ (2012). Next, 25 μL of this solution was serially diluted, plated on BHI agar, and incubated for 48 h at 37°C. Three independent assays were performed for each analysis.

### Knoop Hardness test

Surface microhardness of specimens was measured with a microhardness tester (Shimadzu HMV-2000 Micro Hardness Tester; Shimadzu Corporation, Kyoto, Japan), under a static load (Knoop) of 50 gF for 5 sec. Five indentations were performed on the top surface of the material at 500 μm distance from each other. A mean of the microhardness was obtained for each specimen.

### Fluoride release

Specimens were individually placed in a polystyrene tube containing 4 mL of deionized water, and were left to agitate (TE-420 Orbital shaking table; Tecnal, Piracicaba, SP, Brazil) at room temperature for 24 h. An equal volume of TISAB II (acetate buffer 1.0 M, pH 5.0, containing NaCl 1.0 M and 1.2-cyclohexanediaminetetraacetic 0.4%) was added to the tubes. Specimens were washed with deionized water spray, dried with absorbent paper, and transferred to new tubes containing 4 mL of deionized water. The solutions collected daily were identified and stored in polystyrene tubes at 4°C. Only the solutions from days 1, 3, 7, 10, and 15 were stored. Fluoride release was measured using a fluoride-specific electrode (Orion 9609-BN, Orion Research, Inc., Beverly, USA) connected to a digital ion-analyzer (Orion 720A, Orion 9609-BN, Orion Research, Inc., Beverly, USA), previously calibrated with standard solutions of 0.0625 to 1 or 1 to 16 mg F^−^/mL in TISAB II, and expressed in mg F^−^/cm^2^.

### Clinical procedures and dentin sampling

**Figure 1:**
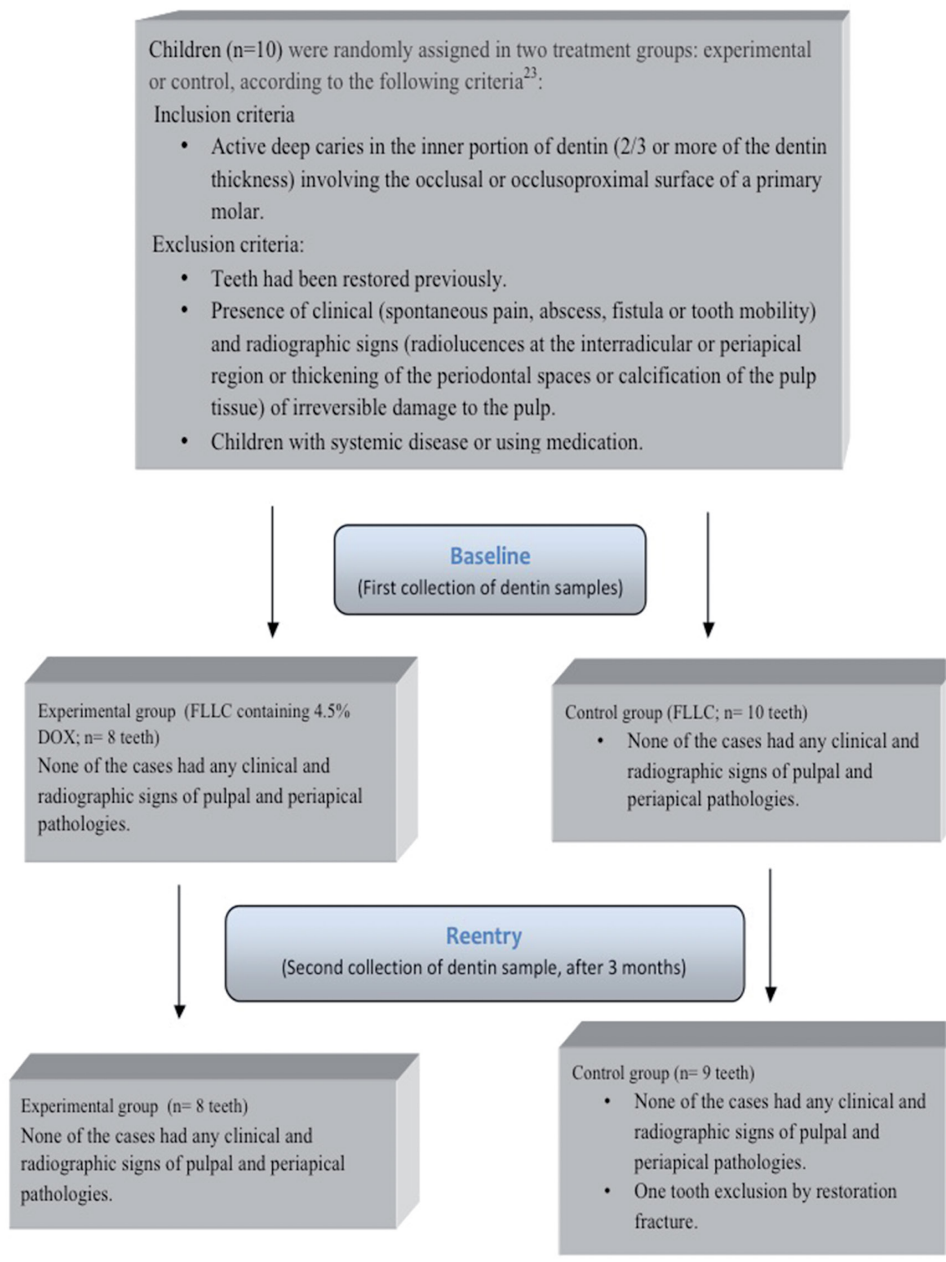
Flow diagram detailing child recruitment, treatment, and follow-up (3 months) of the study

This randomized clinical trial was set in the pediatric clinic of the Piracicaba Dental School, University of Campinas (FOP-UNICAMP), Piracicaba, SP, Brazil, and was approved by the Ethics Committee on Research Involving Human Subjects of the same institution, under protocol number 031/2008. The study was registered in the International Clinical Trial Registry (ClinicalTrials.gov), under identification number NCT02168374. A signed informed consent form was obtained from the legal guardians of each child. Sample for this pilot trial consisted initially of 18 primary molars from 10 children of both genders, aged 4-8 years, based on a previous study^7^. One tooth was excluded from the study because the provisory restoration was lost and 17 teeth comprised the final sample. Details of child recruitment, including inclusion and exclusion criteria, treatment, and follow-up are listed in the flow diagram (Figure 1). Clinical evaluation of pulp vitality was assessed by the presence of clinical signs (pain, abscess, fistula, and abnormal mobility) and, when necessary, in teeth with suspect of irreversible pulpitis, palpation and percussion were performed. Radiographic signs of irreversible pulpitis (widening of periodontal ligament or periapical/interradicular lesions) also completed the clinical diagnosis. No electric or thermal pulp tests were performed in children.

Clinical procedures and dentin sampling were performed by the same investigator (ARFC), in two sessions, following the steps described by Castilho, et al.^23^ (2013). Briefly, at the first session, after bitewing radiograph, anesthesia and rubber dam isolation were performed in the children. Operative area was cleaned by dental prophylaxis and anti-sepsis with 0.2% chlorhexidine. Cavity preparation consisted in removing carious tissue from the cavosurface angle and surrounding walls, and superficial carious dentin from pulp walls with sterile round steel burs at low speed and sterile spoon excavators. After washing and air-drying the cavities, an initial collection (baseline) of the carious dentin was sampled from the mesial portion of the cavity floor using a standard instrument, as previously described^23^. Then, sample was inserted into 0.9% NaCl (5 mL) and the pulpal wall was entirely covered with one of the randomly experimental liner materials: 1) FLLC containing 4.5% DOX or 2) FLLC (control group). DOX concentration was chosen based on the *in vitro* results obtained by Castilho, et al.^16^ (2012) and this study. The cavities were then temporarily restored using a conventional glass ionomer cement-GIC (Ketac Molar, 3M ESPE, Seefeld, Bavaria, GE). Within 3 months after the initial treatment, the teeth were submitted to clinical and radiographic examinations to determine signs and symptoms of pulp vitality. The restorative and liner materials were carefully and completely removed and new dentin sample was collected. When necessary, the remaining softened carious dentin was removed, and the teeth were then restored with a light-cured composite resin (Opallis, FGM, Joinvile, SC, Brazil) using a bonding system, Scotchbond Multi-Purpose (3M ESPE, St. Paul, MN, USA), after a new placement of the initial liner material.

### Clinical recordings

Before all dentin collections, the dental cavities were copiously washed and carefully air dried, and the color, consistency, and wetness of the carious dentin were blindly evaluated by a second investigator (CD), based on the following criteria: dentin consistency: 0 = hard (similar to normal dentin); 1 = leathery (dentin spoon removes carious tissue when forced); 2 = soft (tissue easily removed by a dentin spoon); dentin color: 0 = yellow; 1 = light brown; 2 = dark brown; dentin humidity: 0 = dry; 1 = humid^7^ ^,^ ^23^.

### Microbiological procedures

For cultivation of mutans streptococci, dentin samples were immersed in 0.9% NaCl (5 mL) and homogenized in a tube agitator (Vortex, Phoenix AT 56, Munising, MI, USA) for 1 min. After serial dilutions (10^−1^-10^−6^) of this suspension, 25 μL of the dilutions were seeded in triplicate onto bacitracin (0.2 IU/mL) Mitis Salivarius agar for the growth of mutans streptococci. Plates were incubated at 37°C for 48 h in 5% CO_2_. After incubation, the number of colony-forming units per milliliter (CFU/mL) was counted.

### Statistical analysis

Statistical analysis showed heterogeneous data, and the analysis of variance (one-way ANOVA) was done considering the factors: material and log_10_ (CFU/ mL) or Knoop hardness values or mg F^−^/cm^2^ and the interaction, followed by Tukey's test. Wilcoxon's nonparametric test was used to compare the differences in consistency, color, and wetness of the dentin, before and after partial caries removal. Mutans streptococci counts were compared between the samples collected at baseline and after 3 months within each material group using the same test. Medians and ranges of bacterial counts were expressed as log_10_ (CFU/mL+1). The constant 1 was added to the CFU count, since many samples showed zero CFUs after the experimental period. Complementary Mann-Whitney tests were applied to identify differences among the materials. For statistical analysis, the SPSS version 17.0 software (SPSS Inc., Chicago, IL, USA) was used and the significance level was set at 5%.

## Results

Data from biofilm assays, microhardness, and fluoride release are shown on Table 1. The means and standard deviations of bacterial counts are expressed as log (CFU/mL) after the biofilm assays. Within 24 h, all DOX concentrations reduced the number of bacteria adhered on the GIC, however, there was no statistical difference between the 3 and 4.5% DOX. Groups have similar values of Knoop hardness, without any statistical significant difference among them, showing that the incorporation of DOX in the tested concentrations did not affect the surface microhardness of the cement. Fluoride release was not compromised with the addition of DOX. No statistical difference among the groups was observed for the periods evaluated.

**Table 1:** Summary and statistical comparisons for all *in vitro* assays (mean ± SD)

						Bacterial counts (biofilm)	Surface Hardness
		Fluoride Release (mg F-/mL)				Log (UFC/mL)	(N)
	Day 1	Day 3	Day 7	Day 10	Day 15		
FLLC	11±0.45	1.8±0.078	1.1±0.06	1.1±0.06	1.2±0.07	8.06±0.89a	24.97±2.89
FLLC + 1.5% DOX	10.2±0.38	1.7±0.08	1.1±0.03	1.3±0.08	1.3±0.04	7.08±0.47b	25.16±1.16
FLLC + 3% DOX	9.9±0.55	1.6±0.11	1±0.05	1.2±0.11	1.2±0.07	5.97±0.55c	23.25±1.93
FLLC + 4.5% DOX	9.9±0.41	1.6±0.03	1±0.03	1.1±0.1	1.2±0.04	6.08±0.80c	23.15±3.33

* Different lower case letters in the columns show statistical difference among the groups of materials for bacterial counts, according to ANOVA and Tukey tests (p<0.05). There was no statistical difference among the groups of materials, considering fluoride release and surface hardness, according to ANOVA and Tukey tests (p>0.05).

Based on initial periapical radiograph, all teeth had deep dentin carious lesions. Thirteen out of 18 teeth had caries-active lesions involving only the occlusal surface, while 5 out of 13 teeth showed one approximal surface also involved. Eight teeth were lined with FLLC containing 4.5% DOX and 10 with FLLC. During the trial period, none of the cases had any clinical symptoms (sensitivity to cold, heat, or sweets or spontaneous pain and pressure) and radiographic signs of pulpal and periapical pathologies. There was one case of restoration fracture that required a new filling, and it was excluded from the study. The median/ range of the scores for the clinical and microbiological evaluations, recorded at the baseline and re-entry, are shown in Table 2. No statistical difference was evidenced for any clinical condition evaluated in the experimental and control groups, when the first and the second collections were compared. The remaining dentin in the cavities became harder and dry after 3 months. Regarding the microbiological observation, there was no statistical difference between the groups at baseline (p<0.05). After trial period, there was a significant reduction in bacterial counts with complete absence of *mutans streptococci* counts only for the experimental group.

**Table 2:** Median and range (minimum-maximum) of clinical scores determined at baseline and reentry (after 3 months) of partial caries removal

			Median(range)	
		Material	First Collect (Baseline)	Second Collect (reentry)
	Consistency	FLLC	2(2-2)^Aa^ *	1(0-2)^Ab^
		FLLC + 4.5 % DOX	2(2-2)^Aa^	0(0-0)^Bb^
Dentin condition	Color	FLLC	0(0-0)^Aa^ *	1(0-1)^Ab^
		FLLC + 4.5 % DOX	0(0-0)^Aa^	1(0-1)^Ab^
	Humidity	FLLC	1(1-1)^Aa^ *	1(1-1)^Aa^
		FLLC+ 4.5 % DOX	1(1-1)^Aa^	1(1-1)^Aa^
Microorganism	*mutans streptococci*	FLLC	4.77(4.53-4.8)^Aa^ *	3.75(3.65-5.86)^Aa^
		FLLC+ 4.5 % DOX	4.16(2.76-6.14)^Aa^	0(0-0)^Bb^

* For each material and collect, median (range) followed by: Same lowercase letters in the rows are not statistically different, according to Wilcoxon and Mann-Whitney test (p>0.05). Same uppercase letters in columns are not statistically different, according to Wilcoxon and Mann-Whitney test (p>0.05)

## Discussion

This is the first study to describe the effect of experimental RMGIC liner containing doxycycline hyclate on *in vivo* caries lesions. In addition, more physical and biological properties of that experimental cement were explored. The biological and mechanical findings previously^16^ obtained and the results of this study might help to highlight the use of this material for arresting caries.

Although few studies evaluated the addition of antibiotics to GIC at the time, the results are promising. *In vitro* studies have evidenced improved antibacterial activity against oral pathogens, without influencing the biological and physical properties of the GIC^15^ ^,^ ^16^ ^,^ ^18^. Our research group observed no harmful effect of the addition of different concentrations of DOX on odontoblast cells and compressive and diametral tensile strength of the RMGIC^16^. This study confirms that this combination did not also interfere on the microhardness and fluoride release of the experimental material, besides improving the protection of the material surface against S. *mutans* biofilm formation. Prabhakar, et al.^18^ (2013) added an antibiotic mixture (ciprofloxacin and metronidazole) to GIC and observed that antibiotics at one percent enhance the antibacterial activity and fluoride release of a conventional GIC, without affecting the shear bond strength and microleakage. Contrary to our study that up to 4.5% DOX did not interfere on any tested *in vitro* RMGIC property, Yesilyurt, et al.^15^ (2009) and Prabhakar, et al.^18^ (2013) found that the incorporation of antibiotics into conventional GIC in concentrations above 1.5-2% caused significant alterations in the mechanical properties. It has been considered that different kinds of materials mixed with different antibiotics can perform differently. No study analyzed the microhardness of the RMGIC containing antibiotic to compare with the current data.

The reason for choosing the FLLC for this study is based on its lower inhibitory activity against cariogenic bacteria^7^, but biocompatibility when applied directly on cell culture^27^, possible for its lower HEMA release compared to Vitrebond^28^. The addition of DOX to FLLC considerably increased the antibacterial activity of the cement against S. *mutans* when compared with the RMGIC alone, considering the *in vitro* and *in vivo* methods. One reason for this improvement in the antibacterial activity is the release of doxycycline from glass ionomer cement. Yesilyurt, et al.^15^ (2009) evaluated the release of antibiotics from glass ionomer cements using high performance liquid chromatograph (HPLC) in 24 h and 7 days. The authors observed an increase in the release of antibiotics, including a derivative of tetracycline (minocycline), such as doxycycline, with increase of concentration and time of evaluation.

The absence of mutans streptococci counts after 3 months of partial caries removal showed the microbiological effectiveness of the experimental material. Interestingly, a similar result was only observed by Maltz, et al.^29^ (2002) after the experimental period of 6 months, when all dentin samples exhibited no growth of *S. mutans* or lactobacilli after partial caries removal using a calcium hydroxide liner. Other studies also had encouraging results, testing the antibiotic mixture (metronidazole, ciprofloxacin, and cefaclor) added to GIC after partial caries removal. They evidenced more than a 98% mutans streptococci reduction^13^ and enhanced clinical/radiographic success after 1, 3, 6, and 12 months of partial caries removal procedures when compared to control without antibiotics.^17^

In this study, the clinical effectiveness of DOX added to the RMGIC liner was substantiated by the absence of symptoms and radiographic signs of pulpal and periapical pathologies, indicating pulp vitality. In addition, after partial caries removal, the remaining dentin was darker and harder compared to the first collection (baseline), corresponding to the clinical aspects of arrested caries lesion progression^30^.

Based on the results of this study and considering the short period of evaluation and its limitations, the inclusion of doxycycline into RMGIC maximizes the antibacterial effect of RMGIC, without jeopardizing two important properties of the dental material: fluoride release and surface microhardness. These potential benefits have significant implications for clinical application of RMGIC containing doxycycline as a liner in a one-step incomplete caries excavation to maintain vitality of deeply carious teeth and reduce costs by avoiding cavity reopening. It may be concluded that RMGIC containing doxycycline should be suggested as a novel therapy for treatment of deep caries lesions. However, further *in vitro* assays, including the assessment of doxycycline release and other GIC properties, in addition to long-term clinical trials, should be performed.
